# Inhibitors of telomerase and poly(ADP-ribose) polymerases synergize to limit the lifespan of pancreatic cancer cells

**DOI:** 10.18632/oncotarget.19410

**Published:** 2017-07-20

**Authors:** Katrina M. Burchett, Asserewou Etekpo, Surinder K. Batra, Ying Yan, Michel M. Ouellette

**Affiliations:** ^1^ Department of Biochemistry, University of Nebraska-Lincoln, Lincoln, NE, USA; ^2^ Department of Epidemiology, University of Nebraska Medical Center, Omaha, NE, USA; ^3^ Department of Biochemistry and Molecular Biology, University of Nebraska Medical Center, Omaha, NE, USA; ^4^ Department of Radiation Oncology, University of Nebraska Medical Center, Omaha, NE, USA; ^5^ Department of Internal Medicine, University of Nebraska Medical Center, Omaha, NE, USA

**Keywords:** pancreatic cancer, telomerase, telomere, imetelstat, poly(ADP-ribose) polymerase

## Abstract

Imetelstat (GRN163L) is a potent and selective inhibitor of telomerase. We have previously reported that GRN163L could shorten telomeres and limit the lifespan of CD18/HPAF and CAPAN1 pancreatic cancer cells. Here, we examined the effects of GRN163L on two other pancreatic cancer cell lines: AsPC1 and L3.6pl. In both lines, chronic exposure to GRN163L led to an initial shortening of telomeres followed by a stabilization of extremely short telomeres. In AsPC1 cells, telomere attrition eventually led to the induction of crisis and the loss of the treated population. In L3.6pl cells, crisis was transient and followed by the emergence of GRN163L-resistant cells, which could grow at increasing concentrations of GRN163L. The Shelterin complex is a telomere-associated complex that limits the access of telomerase to telomeres. The telomerase inhibitory function of this complex can be enhanced by drugs that block the poly(ADP-ribosyl)ation of its TRF1 and/or TRF2 subunits. Combined treatment of the GRN163L-resistant L3.6pl cells with GRN163L and 3-aminobenzamide (3AB), a general inhibitor of poly(ADP-ribose) polymerases, led to additional telomere shortening and limited the lifespan of the resistant cells. Results from this work suggest that inhibitors of telomerase and poly(ADP-ribose) polymerases can cooperate to limit the lifespan of pancreatic cancer cells.

## INTRODUCTION

Pancreatic cancer is the fourth leading cause of cancer-related deaths in the Western world. The disease lacks early diagnostic and therapeutic modalities. The tumors, which develop silently in the ducts of the pancreas, are almost always detected in advanced stages, when curative treatments are not possible. The net result is a 5-year survival rate of just 7%, one of the worst among human cancers. In the United States alone, 48,960 patients are estimated to have been diagnosed with the disease in 2015, and an estimated 40,560 patients were predicted to die from it [1]. The majority of pancreatic cancer cases are pancreatic ductal adenocarcinomas, which present themselves as highly invasive tumors consisting of malignant pancreatic ducts embedded in an abundant desmoplastic stroma [2, 3]. The only curative option for these patients is the surgical resection of the tumor [3]. Unfortunately, just 20% of these patients are eligible for the surgery, [3] and for those who get the procedure, the 5-year survival rate is still of only 20% [3]. These statistics show the critical need for novel and improved therapies to treat these patients, as well as prevent the recurrence of the disease. To block the re-growth of residual tumor cells following standard therapy, telomerase inhibitors have been suggested to be particularly well-suited [4, 5]. To assess the effects of telomerase inhibition in pancreatic cancer, in this study, we have characterized the effects on cultured pancreatic cancer cells of long-term exposure to a pharmacological inhibitor of telomerase, GRN163L [6].

Telomerase is the enzyme that maintains telomeres, which are structures that cap and protect the ends of linear chromosomes. Human telomeres are composed of simple (TTAGGG)_n_ DNA repeats and of their associated proteins [7, 8]. This capping structure protects the ends of chromosomes from nucleolytic degradation, end fusions, and from being sensed as double-stranded (ds) DNA breaks, a form of damaged DNA [8, 9]. Telomeres shorten with each cell division because of the inability of the replication machinery to fully replicate the ends of linear DNA molecules, and this attrition limits cellular lifespan [10]. Telomerase can compensate for this loss and extend the lifespan of human cells by the re-elongation of their telomeres. The enzyme is expressed early during human development but is later repressed, so that the activity become undetectable in most somatic tissues after birth [11, 12], including the pancreas [13–15]. Normal human tissues either lack telomerase entirely, as in the case of the pancreas, or express the enzyme at very low level of activity. In contrast, high levels of telomerase activity are commonly detected in cancer specimens. This is the case in more than 85% of human cancers [16], including pancreatic ductal adenocarcinomas [13–15]. Most importantly, this aberrant expression of telomerase is needed for cellular immortality, a hallmark of cancer. In cancer cells expressing telomerase, continuous inhibition of the enzyme results in telomere shortening, which in turns limits the lifespan of the cells [6, 17–20]. Once sufficient telomere shortening has occurred, the treated cancer cells begin to experience senescence and/or apoptosis, depending on the cell type. In cells with functional DNA damage checkpoints, senescence is induced once the shortest telomere has become uncapped and recognized as a ds-DNA break [4, 5]. Cells experiencing senescence are viable but can no longer divide. In cancer cells with dysfunctional checkpoints, signals from uncapped telomeres are ignored, which then allows the cells to keep on dividing until crisis is induced due to terminal telomere shortening. Crisis is a non-viable state [4, 5] brought about by recurrent cycles of telomere end fusions, anaphase bridges, and breakage of the chromosomes [21]. But whether the ultimate response to chronic telomerase inhibition is senescence or crisis, the end result is expected to be tumor growth inhibition. Telomerase is an attractive therapeutic target because it is almost universally expressed in cancer cells, but lacks from most normal tissues. However, the delay needed for the targeted cancer cells to lose sufficient telomeric DNA before senescence or crisis are induced is a potential drawback to the therapeutic use of telomerase inhibitors. This delayed response prevents their use as first line therapy for cancer, but this delay also makes them particularly well adapted to block the regrowth of residual disease following conventional treatments [4, 5], since the growth inhibitory activity of these inhibitors are expected to increase the more the residual cancer cells divide.

Human telomerase is made of two essential subunits: the hTERT protein (human Telomerase Reverse Transcriptase) and hTR, a small nuclear RNA (human Telomerase RNA). hTERT confers catalytic activity whereas hTR includes a sequence (5′-CUAACCCUAA-3′) that serves as template for the production of telomeric DNA [5, 22]. Telomerase's substrate is the telomeric single-stranded 3′-overhang that terminates the end of all telomeres. The enzyme is a reverse transcriptase that uses hTR as template to synthesize telomeric DNA repeats onto the ends of the 3′-overhangs. For the purpose of inhibiting telomerase, the template region of hTR offers an accessible target for oligonucleotidic inhibitors [5, 22, 23]. Oligonucleotides engineered to hybridize with this area of hTR have successfully been used to block telomerase [24, 25]. GRN163L, developed by Geron Corp. (Menlo Park, CA), is a second generation oligonucleotidic inhibitor of telomerase [18]. Also known as imetelstat, GRN163L is a N3′ > P5′ thio-phosphoramidate oligonucleotide designed to hybridize with the template region of hTR. In GRN163L, the N3′ > P5′ thio-phosphoramidate oligonucleotide is conjugated to a 5′-terminal palmitoyl group to facilitate cellular uptake (5′-palmitate-TAGGGTTAGACAA-NH_2_-3′). At nanomolar concentrations, the inhibitor can block telomerase in a wide range of cancer cell lines [18]. In subsequent reports, continuous exposure to GRN163L limited the lifespan of cancer cells from a variety of tumor types, including glioblastoma [26], multiple myeloma [27] Barrett's esophageal adenocarcinoma [28], breast [29, 30], lung [31], liver [32], and pancreatic [6] cancers. In mice, the inhibitor also reduced the growth of tumor xenografts formed by the implantation of human cancer cells [26–28, 30–32]. In human clinical trials, GRN163L has shown promise in patients with multiple myeloma (NCT01242930, NCT00718601, and NCT00594126) and myeloproliferative neoplasms, including essential thrombocythemia and myelofibrosis (NCT01731951, NCT02426086, NCT01243073, and NCT02598661) [33–35].

A second approach to shorten telomeres in cancer cells is to target components of the Shelterin complex. At the telomere, this complex binds with high-affinity to sites at the base of the telomeric 3′-overhang [36]. At these locations, the complex contributes to telomere capping and limits the access of telomerase to its substrate, the telomeric 3′-overhang [37]. The Shelterin complex contains up to six core components: TRF1, TRF2, RAP1, TIN2, TPP1, and POT1. Three of these factors associate directly with telomeric DNA, either in its single-stranded (POT1) or double-stranded (TRF1, TRF2) form [37]. Importantly, the DNA-binding activities of two of these factors, TRF1 and TRF2, is regulated by the activities of poly(ADP-ribose) polymerases (PARPs). PARPs are enzymes that transfer chains of poly(ADP-ribose) to target proteins as a mean of regulating their biochemical activities [38]. PARP1 and PARP2 associate with TRF2 and promote its poly(ADP-ribosyl)ation (or parsylation), a post-translational modification that blocks the DNA-binding activity of TRF2 [39, 40]. TRF1 can also be modified by the PARP enzymes TNKS1 and TNKS2, and this parsylation of TRF1 inhibits its DNA-binding activity and promotes its ubiquitin-mediated degradation [41–43]. In telomerase-expressing cancer cells, the overexpression of TNKS1 reduces the level of TRF1 at the telomere, thereby giving telomerase an increased access to telomeres and resulting in a gradual lengthening of telomeres [44]. In three reports, telomeres were shortened by prolonged exposure to the general PARP inhibitor 3-aminobenzamide (3AB), used either alone [45, 46] or in combination with the telomerase inhibitor MST-312 [47]. In a third report, the effect of general PARP inhibition on telomere maintenance was recapitulated by the knockdown and selective inhibition of PARP1, thereby implying a predominant role for PARP1 in controlling the activities of the Shelterin complex [45]. PARP inhibitors have not yet been tested in combination with GRN163L, the first telomerase inhibitor to enter phase II clinical trials.

We have previously reported on the effects of chronic GRN163L on the lifespan of CAPAN1 and CD18/HPAF pancreatic cancer cell lines [6]. In both lines, GRN163L led to a rapid shortening of telomeres, followed by the stabilization of terminally short telomeres. Both cell lines eventually succumbed to crisis and cultures were lost to the induction of senescence and apoptosis [6]. Here, we have examined the effects of chronic GRN163L exposure in two additional lines of pancreatic cancer cells, AsPC1 and L3.6pl. Whereas the AsPC1 cells responded to chronic GRN163L with terminal telomere shortening, induction of crisis, and loss of the culture, the L3.6pl cells became resistant to increasingly higher concentrations of the drug. Here, we describe the properties of these GRN163L-resistant cells, including their critically short telomeres, absence of ALT mechanism, and decreased response to GRN163L. We also show that the combined treatment of these GRN163L-resistant L3.6pl cells with GRN163L and general PARP inhibitor 3-aminobenzamide (3AB) led to additional telomere attrition and was sufficient to induce crisis and limit the lifespan of the resistant cells. These results suggest that inhibitors of telomerase and poly(ADP-ribose) polymerases can cooperate to limit the lifespan of pancreatic cancer cells.

## RESULTS

### Effects of chronic GRN163L exposure on the lifespan of AsPC1 and L3.6pl cells

We have previously reported that GRN163L shortens telomeres and limits the lifespan of CD18/HPAF and CAPAN1, both of which expressing low levels of telomerase [6]. In this report, we have examined the effects of the drug on two other pancreatic cancer cell lines, both of which expressing higher levels of telomerase activity: AsPC1 and L3.6pl. As we have done previously [6], the two lines were cultivated in the presence of no drug (CTR), 1 μM GRN163L (GRN), or 1 μM of a mismatched oligo (MIS). The mismatched oligo was identical to GRN163L, except for four base mismatches designed to prevent binding to hTR (5′-palmitate-TAGGTGTAAGCAA-NH_2_-3′; mismatches underlined). GRN163L was added fresh every 2–3 days. Once a week, cells were counted and re-plated at a lower density to keep them in log phase growth. Excess cells were made into frozen stocks or were collected for telomere length analysis.

**Figure 1:**
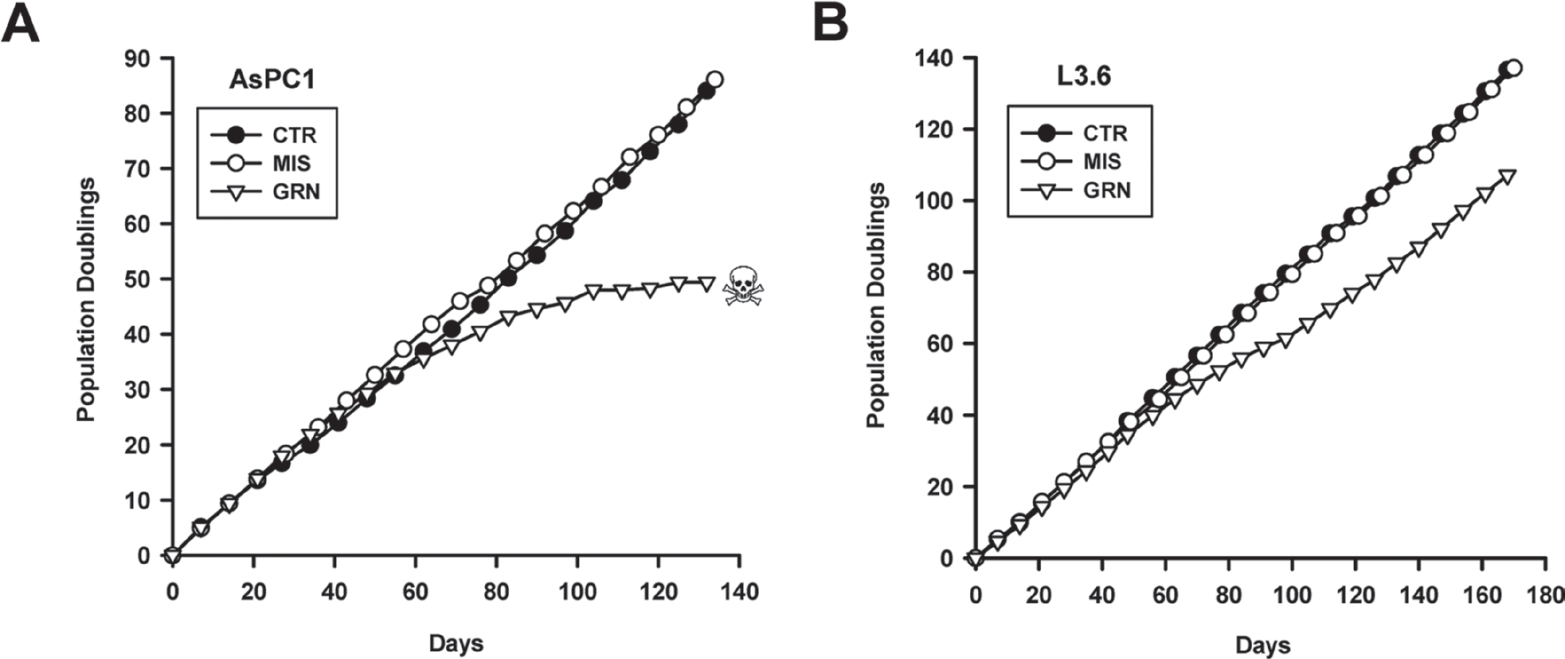
Effects of continuous GRN163L exposure on lifespan Each line was continuously treated with no drug (PBS vehicle; CTR), GRN163L (1 μM; GRN), or the Mismatch oligo (1 μM; MIS). Cells were given fresh drugs every 2–3 days. Once per week, cells were counted and replated. Every other week, excess cells were set aside for either telomere length analysis or frozen down. Growth curves show the number of population doublings done as a function of time for the AsPC1 (**A**) and L3.6pl (**B**) cells. Skull and bone denotes loss of the culture to crisis.

**Figure 2:**
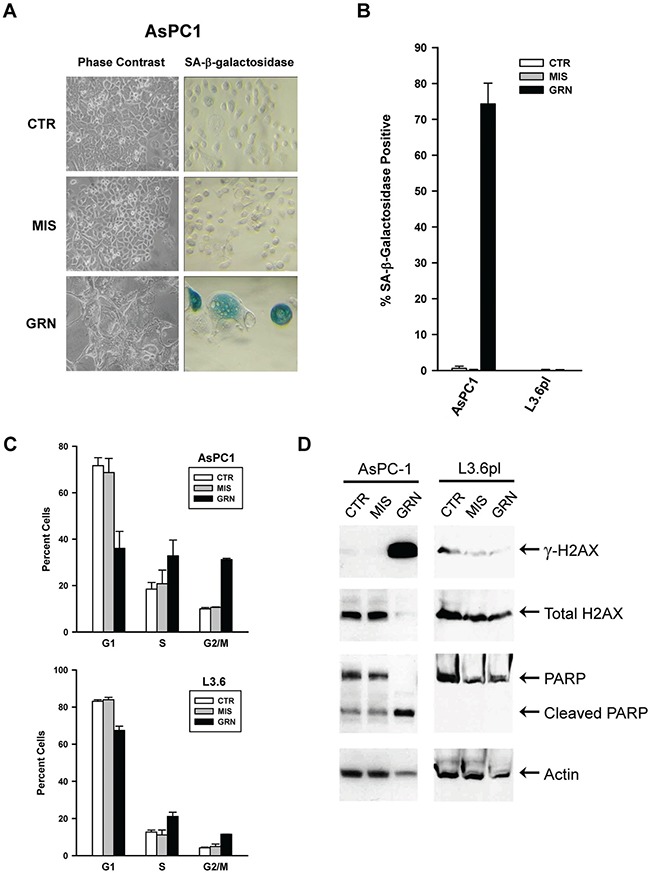
Markers of senescence, apoptosis, and DNA damage response in the GRN163L-exposed AsPC1 and L3.6pl cells At the end of the curves presented in Figure 1A and 1B, cells treated with no drug (CTR), mismatch oligo (MIS) and GRN163L (GRN) were analyzed for evidence of senescence, apoptosis, and DNA damage response. (**A**) Histological analysis of AsPC1 cells. Phase contrast images (Left panels). Histochemical staining reveals the presence of SA-β-galactosidase activity in the GRN163L-exposed AsPC1 cells, as shown by the presence of insoluble blue pigments (Right panels). (**B**) Percent of cells in each sample that were marked by SA-β-galactosidase activity (Mean ± S.D., *n* = 3). (**C**) Cell cycle analysis of the GRN163L-treated and -untreated cells. Cells were stained with propidium iodide and analyzed by flow cytometry. Two measurements were made at a one week interval for the AsPC1 and L3.6pl cells (Mean ± S.D., *n* = 2). Percent cells in the G1, S and G2/M phases of the cell cycle is shown (G1 + S + G2/M = 100%). (**D**) Western blot analysis of the GRN163L-treated and -untreated cells. Samples were analyzed with antibodies against histone H2AX, phosphorylated H2AX (γ-H2AX), PARP1 and actin.

Figures 1A and 1B show the impacts of continuous GRN163L on the division and lifespan of AsPC1 and L3.6pl cells, respectively. In both cell lines, control populations exposed to either no drug (CTR) or to the mismatched oligo (MIS) grew at the same constant rate over the course of the experiment. In their first 4–8 weeks of treatment, cells exposed to GRN163L (GRN) grew at the same rate as the control populations (CTR, MIS). But thereafter, the GRN163L-treated cells began to experience decreased proliferation. In the AsPC1 cells, proliferation of the GRN163L-treated cells continued to decline until the culture was lost to crisis (Figure 1A). Crisis in this population was characterized by the gradual accumulation of floating cells, suggestive of cell death, and by the presence of adherent cells with the flat and enlarged morphology of senescent cells (Figure 2A; data not shown). Loss of the GRN163L-treated AsPC1 cells occurred after 49 divisions done in the presence of the drug. In the case of the GRN163L-treated L3.6pl cells, the decline in proliferation was only transient (Figure 1B). The GRN163L-treated L3.6pl cells exhibited a slowing of their growth rate after 60 days of growth. But soon after this initial crisis, a sub-population eventually emerged that was no longer inhibited by the drug. By day 112 (PD 70), these cells had fully recovered to exhibit growth rates that were almost identical to that of the control populations (CTR, MIS). At day 168 (PD 107), these cells were still dividing in spite of their continuous exposure to 1 μM GRN163L.

At the end of their respective growth curves (Figures 1A and 1B), the different populations were analyzed for evidence of senescence and apoptosis. As the GRN163L-treated AsPC1 approached crisis, an increase in attached cells displaying the flat and enlarged phenotype of senescent cells was detected. Staining for the presence of SA-β-galactosidase activity, a marker of senescent cells [48], further established that these cells had senesced (Figures 2A and 2B). A substantial proportion of cells exhibiting SA-β-galactosidase activity was noted in the GRN163L-exposed AsPC1 but not in their corresponding controls (CTR, MIS) or in any of the L3.6pl samples. Cell cycle analysis was also performed with cells collected at the end of the growth curves. The analysis revealed an increased in the fraction of cells at the S and G2/M phases in the GRN163L-treated populations (GRN) compared to the control populations (CTR, MIS)(Figure 2C). These GRN163L-produced alterations were observed in both the AsPC1 and L3.6pl cells. A large increase in cells with a sub-G1 DNA content was also noted in the GRN163L-exposed AsPC1, but not in their corresponding control populations (CTR, MIS) or in any of the L3.6pl samples (Supplementary Figure 1). Cells with a sub-G1 DNA content is suggestive of apoptosis, as this form of cell death results in DNA fragmentation [49]. To confirm the presence of apoptotic cells, samples were analyzed for evidence of PARP1 cleavage (Figure 2D). PARP1 (Poly(ADP-ribose) polymerase 1) is a substrate of caspase-3 and is cleaved during apoptosis [50]. As Figure 2D shows, PARP1 was almost completely cleaved in the GRN163L-treated AsPC1, but not in their corresponding controls (CTR, MIS) or in any of the L3.6pl samples (Figure 2D). Collectively, these results demonstrate that chronic GRN163L exposure can limit the lifespan of AsPC1 cells through the concomitant induction of senescence and apoptosis. They are also consistent with the emergence in L3.6pl cells of a subpopulation that resists the effects of chronic GRN163L exposure.

### Effects of continuous GRN163L on the maintenance of telomeres

**Figure 3:**
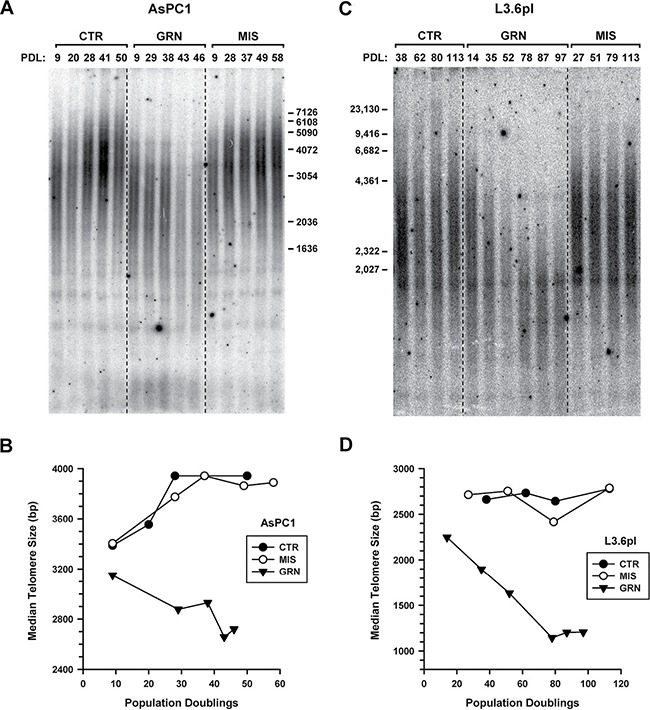
Effects of continuous GRN163L exposure on the maintenance of telomeres Cells were treated as described in Figure 1 with no drug (CTR), mismatch oligo (MIS) or GRN163L (GRN). (**A**, **C**) Southern blot analysis of the length of telomeres. At the indicated population doublings (PDL), genomic DNA was isolated from the GRN163L-treated and -untreated AsPC1 (A) and L3.6pl (C) cells. Genomic DNA was cut with restriction enzymes, separated by electrophoresis, and detected by hybridization to a [^32^P]-(CCCTAA)_4_ probe. (**B**, **D**) Telomere length measurements. Median telomere lengths are presented for the GRN163L-treated and -untreated AsPC1 (B) and L3.6pl (D) cells.

In the AsPC1 cells exposed to no drug (CTR) or to the mismatched oligo (MIS), telomeres became longer during the course of the experiment, from 2.4 to 4.0 kb (Figures 3A, 3B). The reasons for this elongation are still unclear. But in the GRN163L-treated AsPC1 cells, telomeres did not substantially elongate and eventually became increasingly short (Figures 3A, 3B). At PD 46, just before the culture was lost to crisis, telomeres had decreased to a length of 2 kb only. These cells were examined for evidence of telomere dysfunction, using phosphorylated H2AX (γ-H2AX) as an indication of an ongoing DNA damage response [51]. As Figure 2D shows, γ-H2AX was markedly induced in the GRN163L-treated AsPC1 cells (GRN) but not in their corresponding controls (CTR, MIS).

In the L3.6pl populations exposed to no drug (CTR) or to the mismatched oligo (MIS), telomeres were stable over time (Figures 3C, 3D). In the GRN163L-treated L3.6pl cells, telomeres shortened progressively in the time period leading to and throughout crisis (Figure 3C, 3D). However, once cells had emerged that were no longer inhibited by GRN163L (Figure 1B), telomeres became stabilized. In these post-crisis cells (PD > 70), telomeres were exceptionally short (1.2–1.3 Kb range; Figures 3C, 3D) but were not associated with increased γ-H2AX (Figure 2D). These results suggest that in the post-crisis L3.6pl cells, telomeres were kept functionally capped, as to not elicit a DNA damage response in spite of their exceptionally short size.

### Evidence of GRN163L resistance in the post-crisis L3.6pl cells

**Figure 4:**
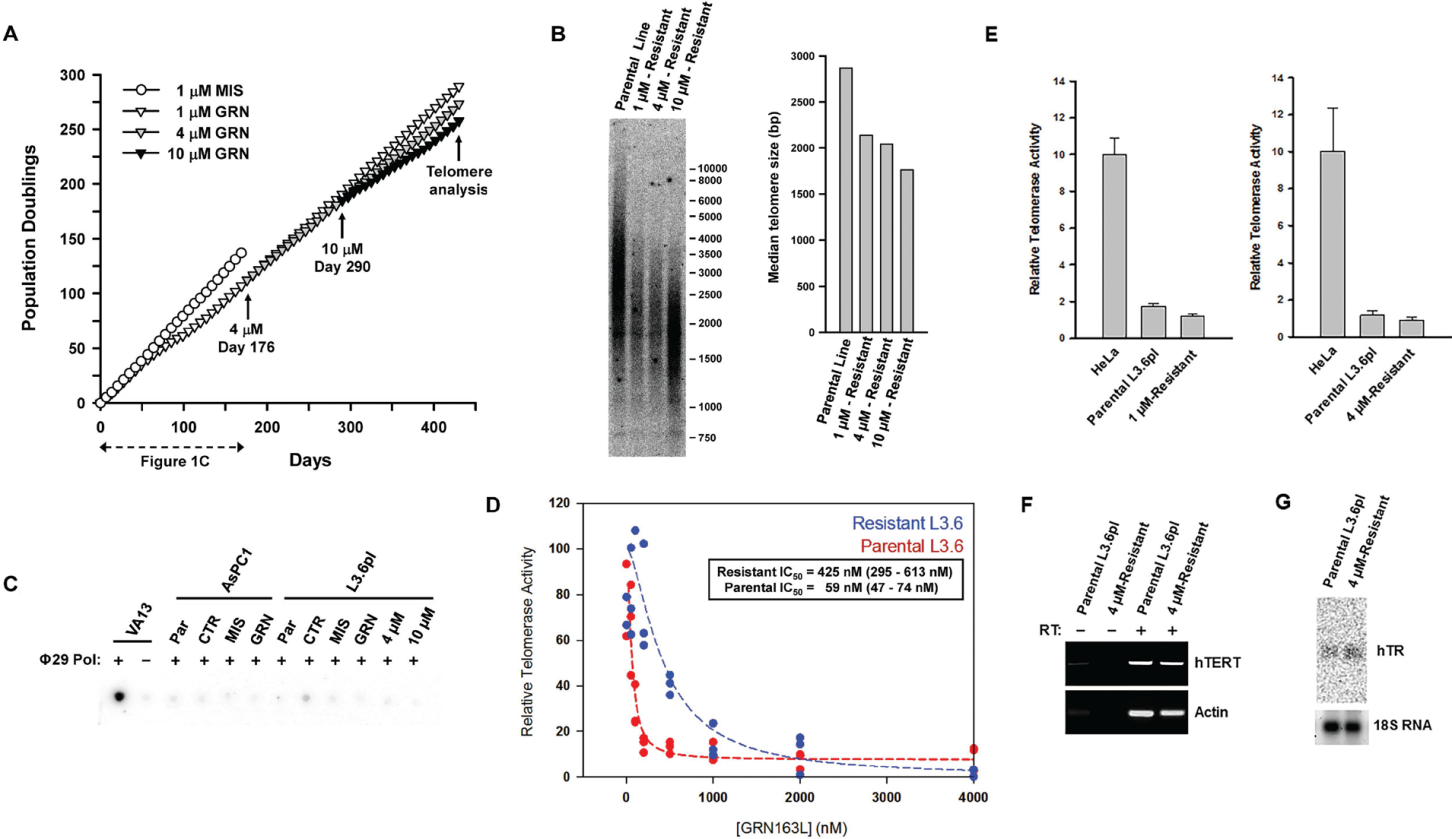
Development of resistance to GRN163L in the L3.6pl cells (**A**) Cultivation of L3.6pl cells at increasingly higher concentrations of GRN163L. The GRN163L-treated L3.6pl cells from day 172 of Figure 1B were initially cultivated in the presence of either a maintenance dose of 1 μM GRN163L or 4 μM GRN163L. At day 290, cells grown in the presence of 4 μM GRN163L were either kept in 4 μM GRN163L or switched to 10 μM GRN163L. The new data not already presented in Figure 1C starts at Day 176. (**B**) At the end of the curve, cells were analyzed for differences in telomere lengths. Median length of telomeres was compared between the parental L3.6pl cells and L3.6pl cells obtained at the end of Figure 4A (1 μM, 4 μM, and 10 μM-Resistant cells). (**C**) Absence of ALT pathway activation in the GRN163L-treated AsPC1 and L3.6pl cells. Samples were analyzed for the presence of C-rich telomeric circles indicative of ALT. VA13 cells, which use ALT to maintain their telomeres, were used as positive control. Reactions were performed in the presence (+) or absence (−) of Φ29 DNA polymerase. Synthesis of the complementary G-rich strand was revealed by hybridization to a [^32^P]-labeled (TAACCC)_4_ probe. For each cell line, samples analyzed included the parental cells (Par) and those harvested at the end of Figures 1A–1B (CTR, MIS, and GRN). Also included were the GRN163L-resistant L3.6pl cells obtained at the end of Figure 4A (4 uM- and 10 uM-resistant). (**D**) Response of the parental and GRN163L-resistant L3.6pl cells to GRN163L. After three weeks of growth in the absence of GRN163L, the 4 μM-resistant L3.6pl cells (from the end of Figure 4A) were tested for their response to GRN163L. Parental L3.6pl cells were used as controls. In triplicates, cells were exposed to increasing concentrations of GRN163L and level of telomerase activity was quantified 24 hours later. Box show the values of the IC_50_ calculated for each sample (value and 95% confidence interval). (**E**) Basal telomerase activity in the parental and GRN163L-resistant L3.6pl cells. Cells adapted to either 1 μM (Left) or 4 μM (Right) GRN163L were cultivated in the absence of the drug for three weeks, after which point basal telomerase activity was measured. Activity is expressed as a percent of the amount of telomerase detected in HeLa cells (Mean ± S.D., *n* = 3). (**F**) Expression of the hTERT gene in the parental and GRN163L-resistant L3.6pl cells. Total RNA from the two lines was reversed transcribed and subjected to PCR with primers specific for hTERT or actin. Reactions were performed in either the presence (+) or absence (−) of reverse transcriptase (RT). (**G**) Level of hTR in the parental and GRN163L-resistant L3.6pl cells. Northern blot analysis was performed with 10 micrograms of total RNA from each of the two lines. Membrane was probed with a random primed [^32^P]-labeled probe against hTR. 18S ribosomal RNA was used to normalize signals for loading variation.

The emergence of an L3.6pl sub-population that could grow and maintain telomeres in the continuous presence of 1 μM GRN163L suggested that these cells had become resistant to the drug. To confirm that this was indeed the case, the post-crisis L3.6pl cells were re-expanded in the presence of increasing concentrations of GRN163L (Figure 4A). In a first step, the emerging cells were switched to culture media containing 4 μM GRN163L. A parallel culture, used as control, was kept in 1 μM GRN163L. As Figure 4A shows, the two cultures grew at the same rate for more than 73 doublings (113 days) to produce superimposable growth curves. In the second step, these 4 μM-resistant cells were then switched to media containing 10 μM GRN163L. As a control for this step, some of the cells were kept in media containing 4 μM GRN163L. As Figure 4A shows, switching the 4 μM-resistant cells to 10 μM GRN163L did result in a modest growth inhibition. But after 73 doublings (141 days) done in the presence of 10 μM GRN163L, cells eventually recovered to achieve growth rates identical to that of the control cells kept in 4 μM GRN163L (Figure 4A). Telomere analysis revealed that by day 430, populations maintained in the presence of GRN163L all had acquired longer telomeres (1.7 to 2.2 kb range; Figure 4B), at least compared to when the cells had just emerged from crisis (1.2 kb; Figures 3C, 3D). Although maintenance at the higher GRN163L concentrations led to telomeres that were shorter (Figure 4B; 10 μM vs. 4 μM vs. 1 μM), these telomeres were still longer than those detected during crisis (1.2 kb; Figure 3C, 3D). Collectively, these results demonstrate that the L3.6pl cells have become resistant to the telomere-shortening effects of continuous GRN163L exposure.

### No evidence of ALT mechanism in the GRN163L-resistant L3.6pl cells

Telomeres in human cancer cells can be maintained by one of two mechanisms: through the activity of telomerase or by the alternative lengthening of telomeres (ALT). The ALT mechanism is associated with the presence of telomeric C-rich DNA circles as well as long and heterogeneous telomeres [52, 53]. To test the possibility that the L3.6pl cells may have become resistant to GRN163L as a result of the activation of the ALT mechanism, we have tested the cells for the presence of telomeric C-rich circles, as described by the Reddel group [54]. In the assay, the circles serve as self-priming templates for the synthesis of G-rich telomeric DNA by the rolling circle DNA polymerase Φ29. Detection of these G-rich strands after Φ29 is then achieved by hybridization to a [^32^P]-(TAACCC)_4_ probe [54]. Cell line VA13, which employs ALT to maintain its telomeres [55], was used as a positive control. Incubation of VA13 DNA with DNA polymerase Φ29, as expected, led to the abundant generation of G-rich telomeric DNA (Figure 4C). This abundant synthesis of G-rich DNA after Φ29 was not observed in any of L3.6pl samples, including DNA isolated from 10 μM GRN163L-resistant cells (Figure 4C). These results, along with the absence of long and heterogeneous telomeres, show that the resistance of L3.6pl cells to GRN163L is not caused by activation of the ALT mechanism.

### Decreased inhibition of telomerase by GRN163L in the GRN163L-resistant L3.6pl cells

Next, we asked if the development of GRN163L resistance was accompanied by a decrease in the response of L3.6pl cells to GRN163L. In dose-response curves, the effect of GRN163L on telomerase activity was compared between the 4 μM-resistant L3.6pl cells (Day 246) and the parental L3.6pl cells (Day 0). Cells were washed, replated, and grown for a week in the absence of GRN163L, long enough to reverse the effects of the drug. The 4 μM-resistant cells, along with the parental L3.6pl cells, were then re-exposed to increasing concentrations of GRN163L (*n* = 3 per dose). Twenty-four hours after GRN163L addition, telomerase activity was quantified by the TRAP telomerase assay, as we have done previously [6]. Expressing telomerase activity as a function of GRN163L concentration produced dose-response curves, which were then fitted to estimate the IC_50_ values of the compound in each of the two L3.6pl samples. As indicated by the results of Figure 4D, inhibiting telomerase in the 4 μM-resistant cells required 7-fold higher concentrations of GRN163L than in the parental L3.6pl cells (IC_50_ of 425 nM versus 59 nM). This higher IC_50_ in the resistant cells compared to the parental cells was observed in 3/3 additional experiments. These results show that in the 4 μM GRN163L-resistant L3.6pl cells, GRN163L inhibits telomerase with markedly reduced potency.

In follow-up experiments, we have investigated the telomerase complex for biochemical alterations that could explain the reduced response of the resistant L3.6pl cells to GRN163L. The direct target of GRN163L is the template region of the human telomerase RNA (hTR, or TERC). Mutations in this template could potentially reduce the response to GRN163L. We have sequenced the hTR RNA expressed in the 4 μM-resistant L3.6pl cells. No evidence of point mutations were detected in 10/10 independently cloned and sequenced hTR molecules (Data not shown). Levels of hTR, hTERT mRNA and basal levels of telomerase activity were also found to be unchanged between the 4 μM-resistant and parental L3.6pl cells (Figures 4E–4G).

### 3-aminobenzamide synergizes with GRN163L to limit the lifespan of GRN163L-resistant L3.6pl cells

Certain members of the PARP family (PARP1, PARP2, TNKS1 and TNKS2) have been reported to parsylate and inhibit the DNA-binding activity of the Shelterin complex, an important negative regulator of telomerase [39–43]. The general PARP inhibitor 3-aminobenzamide (3AB) has previously been used to shorten telomeres in cancer cells [45–47], but the inhibitor has never been tested in combination with GRN163L. In this section, we have tested 3AB as a complementary strategy to shorten telomeres in the GRN163L-resistant L3.6pl cells.

**Figure 5:**
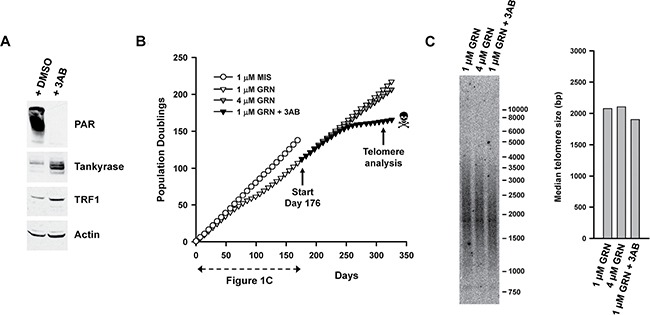
Exposure to 3AB limits the lifespan of GRN163L-resistant L3.6pl cells (**A**) Effects of 3AB on protein parsylation and levels of TRF1 and Tankyrases. Parental L3.6pl cells were exposed to either 3AB (3 mM) or DMSO (Vehicle). Twenty-four hours later, they were analyzed by Western blotting. (**B**) Effects of chronic 3AB on the lifespan of GRN163L-resistant L3.6pl cells. At day 176, the GRN163L-treated L3.6pl cells obtained at the end of Figure 1B were re-cultivated in the presence either 1 μM GRN163L (as maintenance dose), 1 μM GRN163L plus 3 mM 3AB, or 4 μM GRN163L. Cells were given fresh GRN163L every 2–3 days and fresh 3AB twice a week. Growth curves show the number of population doublings achieved as a function of time. Skull and bone denotes loss of the culture. The new data not already presented in Figure 1C starts at Day 176. (**C**) At the end of the curve, cells were analyzed for differences in telomere lengths. Median length of telomeres was compared between the different treatments.

As a first step towards testing 3AB, the effects of 3AB on levels of the Tankyrases (TNKS1 and TNKS2), TRF1 and total parsylated proteins were determined in parental L3.6pl cells. Cells were treated for 24 hours with 3 mM 3AB, a dose previously reported to be synergistic with the telomerase inhibitor MST-312 [47]. In human cells, PARP1 is responsible for the majority of protein parsylation (85%–90%) whereas the remaining activity is predominantly carried out by PARP2 [56]. In L3.6pl cells, exposure to 3 mM 3AB was sufficient to reduce the amount of parsylated proteins to an undetectable level (Figure 5A). This result implies an almost complete inhibition of the activities of PARP1 and PARP2, as the two enzymes are responsible for more than 90% of all protein parsylation [56]. The treatment with 3AB also led to an increase in the level of TRF1 (Figure 5A), as it is expected after inhibition of the Tankyrases (TNKS1, TNKS2). Parsylated TRF1 is unstable and subjected to ubiquitin-mediated degradation. Consequently, TRF1 is stabilized by the inhibition of the Tankyrases [41–43]. Exposure to 3AB also led to an increase in the levels of the Tankyrases (Figure 5A), as one would expect following Tankyrase inhibition since these enzymes are normally destabilized by their own auto-parsylation [57]. Finally, we observed that the activity of telomerase was unaffected by incubation of the cells with 3AB (Data not shown). These results indicated that this concentration of 3AB was sufficient to inhibit the activities of the PARP family members that regulate Shelterin function (PARP1, PARP2, TNKS1 and TNKS2).

Next, we examined the effects of 3AB on the lifespan of the GRN163L-resistant L3.6pl cells. In Figure 5B, the 1 μM GRN163L-resistant cells (GRN163L-treated L3.6pl cells from day 172 of Figure 1B) were grown in medium containing a maintenance dose of GRN163L (1 μM, added thrice weekly) in either the presence or absence of concomitant 3AB (3 mM, added biweekly). Cells expanded in the presence of a higher concentration of GRN163L alone (4 μM, added thrice weekly) were also included as a control. As already described in the previous section, cells exposed to 1 μM and 4 μM GRN163L grew at the same constant rate to produce superimposable curves, with no evidence of crisis (Figures 4A, 5B). In contrast, those grown in the presence of 1 μM GRN163L plus 3AB slowed down and reached a plateau (Figure 5B). After a delay of 38 doublings, these cells eventually became growth inhibited, displayed hallmarks of crisis, and were subsequently lost. Samples collected at the end of the growth curve (day 325) were analyzed for changes in telomere length. While telomeres were of similar length between the 1 μM and 4 μM GRN163L-treated cells, cells treated with 1 μM GRN163L plus 3AB had shorter telomeres – a result consistent with the telomere-shortening effects of PARP inhibitors (Figure 5C). These results show that 3AB can be used to shorten telomeres and limit the lifespan of GRN163L-resistant cancer cells.

## DISCUSSION

In a previous article, we documented the effects of chronic GRN163L exposure in two pancreatic cancer cell lines, CD18/HPAF and CAPAN1 [6]. In both lines, continuous exposure to GRN163L led to telomere attrition, followed by the induction of crisis, and then the loss of the cultures. Here, we tested the effects of chronic GRN163L in two additional lines of pancreatic cancer cells, AsPC1 and L3.6pl. In both AsPC1 and L3.6pl cells, as we have previously reported for CD18/HPAF and CAPAN1 cells, chronic GRN163L led a progressive shortening of the telomeres. In AsPC1 cells, as in CD18/HPAF and CAPAN1, this attrition eventually led to telomere dysfunction, as evidenced by the activation of a DNA damage response, entry into crisis, and loss of the culture. In all three cell lines, crisis was accompanied by evidence of both senescence and apoptosis. In the L3.6pl cells, the telomere shortening produced by GRN163L was not followed by the loss of the culture to crisis. In the GRN163L-treated L3.6pl cells, we saw no evidence of senescence, apoptosis, or activation of a DNA damage response. Instead, we observed the emergence of cells that became resistant to increasingly higher concentrations of GRN163L, up to 10-times the initial dose of the drug. These cells continued to divide with extremely short but stable telomeres in spite of their continuous exposure to GRN163L. This is the first report of a development of resistance to GRN163L. Out of 4 pancreatic cancer cells tested, three responded to GRN163L with the drug resulting in the loss of the cells (AsPC1, CD18/HPAF and CAPAN1). The fourth line eventually developed resistance to the effects of the drug (L3.6pl).

Chronic exposure to GRN163L initially caused the L3.6pl cells to shorten their telomeres and enter a state of crisis, as evidenced by a greatly reduced growth rate (Figure 1B). But eventually, cells emerged from crisis that were no longer inhibited by 1 μM GRN163L. This emerging population had very short telomeres (1.2 kb in length; Figure 3D) that were otherwise adequately capped, as evidenced by the absence of increased H2AX phosphorylation (Figure 2D). By day 430, telomeres in these emerging cells had grown back up to 2.1 kb in length, in spite of the continuous presence of 1 μM GRN163L (Figure 4B). Switching these cells to a 10-fold higher concentration of GRN163L only marginally decreased the length of telomeres to 1.8 kb and failed to limit cellular lifespan (Figure 4A, 4B). Three plausible mechanisms can be envisioned for the resistance of these cells to the telomere-shortening activities of GRN163L: 1) activation of the ALT pathway (Alternative Lengthening of Telomeres), a telomerase-independent mechanism for the maintenance of telomeres; 2) a reduction of the ability of GRN163L to inhibit telomerase; or 3) changes in telomere length regulation that supports telomere maintenance in cells with greatly reduced levels of telomerase activity.

The ALT pathway uses a rolling circle process to grow telomeres without telomerase, and cells that use the ALT pathway tend to have long and heterogeneous telomeres [54]. We saw no evidence of the ALT pathway in the GRN163L-resistant L3.6pl cells. Not only were their telomeres homogenously short, but the presence of C-rich telomeric circles needed to support ALT could not be detected (Figure 4C). On the other hand, we did see changes in the ability of GRN163L to inhibit telomerase in the GRN163L-resistant cells (Figure 4D). Dose-response curves have indeed revealed changes in the response of the cells to GRN163L, with the GRN163L-resistant cells requiring 7-times higher concentrations of GRN163L to inhibit telomerase to the same extent as in parental L3.6pl cells (IC_50_ of 425 nM versus 59 nM). This shift in the response to GRN163L suggests changes in the function of the telomerase complex and/or changes in the cellular uptake, intracellular transport and/or stability of GRN163L. Because the GRN163L-treated cells experienced crisis prior to developing their resistance to GRN163L, it must be that the resistant phenotype has emerged as a consequence of the selection of rare cells with distinct genetic and/or epigenetic alterations. We have examined the possibility that these putative alterations might be involving hTR and hTERT, but found no evidence of point mutation in hTR (Data not shown) or changes in the levels of hTR, hTERT mRNA, or basal telomerase activity (Figures 4E–4G). We have now undertaken RNA-seq analyses comparing the GRN163L-resistant and parental L3.6pl cells to identify mutations in transcripts and changes in gene expression that correlate with GRN163L resistance. These studies are in progress and will require validation before the changes responsible for GRN163L resistance can be identified.

Finally, we cannot exclude the possibility that changes in telomere length regulation may also have taken place that might contribute to the resistance of L3.6pl cells to GRN163L. We have recently reported that in pancreatic cancer cells, exposure to GRN163L leads to a rapid shortening of telomeres followed by a telomere stabilization that delays the progression of crisis. We have previously proposed that this stabilization is a consequence of an auto-regulatory loop involving the Shelterin complex [6]. In our previous report, GRN163L-treated pancreatic cancer cells accumulate extremely short telomeres depleted in the Shelterin subunit TRF2 [6]. This depletion in Shelterin complexes could make these extremely short telomeres an even better substrate for traces of residual telomerase activity, as these complexes normally restricts the access of the enzyme to the telomere. We again observed this initial rapid shortening followed by telomere stabilization in the L3.6pl cells. One possibility might be that Shelterin complexes are already in short supply in L3.6pl cells, thereby allowing these cells to maintain their telomeres with exceedingly low levels of telomerase, including traces of residual telomerase activity that may remain after inhibition by GRN163L. In agreement with this possibility, we show here that drugs designed to increase the abundance of Shelterin complexes can block the continued proliferation of the GRN163L-resistant L3.6pl cells.

The telomerase inhibitory function of the Shelterin complex can be controlled by the parsylation of its TRF1 and TRF2 subunits [39–43]. This parsylation is respectively carried out by the TNKS1/2 and PARP1/2 members of the PARP family, and drugs that block these enzymes have been reported to increase TRF1 and shorten telomeres [45–47, 58]. In L3.6pl cells treated with 3AB, similar changes were observed: TRF1 is increased (Figure 5A) and telomeres are shorter (Figure 5C). When combined with GRN163L, 3AB also contributed to reduce the lifespan of the L3.6pl cells. Neither 3AB alone nor GRN63L alone were sufficient to block the proliferation of the parental L3.6pl cells, but the two drugs combined could limit the lifespan of the parental cells, causing them to experiencing crisis earlier while blocking the development of GRN163L resistance (Supplementary Figure 2). In L3.6pl cells that had already become resistant to GRN163L, the addition of 3AB resulted in the induction of crisis and loss of the culture (Figure 5B). These results are reminiscent of the previously reported synergy between 3AB and telomerase inhibitor MST-312 [47].

3AB limits cellular lifespan when combined with telomerase inhibitors, but the underlying mechanisms have not yet been fully defined. In cells treated with 3AB, TRF1 is increased and this increase could potentially boost the activity of at least two complexes controlling lifespan and proliferation: TRF1-containing Shelterin complex and TRF1-containing SA1 Cohesin complex [59, 60]. An increase in TRF1-containing Shelterin complexes would be expected to decrease the access of residual telomerase to the telomere, thereby causing telomere attrition and reducing cellular lifespan. This first mechanism has already been implicated in the synergy between 3AB and MST-312 [47]. That same mechanism is likely to be involved here also, given that telomeres were shortened by 3AB in the GRN163L-resistant L3.6pl cells (Figure 5B). Yet, one still cannot exclude a potential role played by the other complex, the TRF1-containing SA1 Cohesin complex. A change in the activity of this complex could also affect cellular lifespan, but in different ways. In the S phase, the TRF1-containing SA1 Cohesin complex helps maintain cohesion between telomeres of sister chromatids [61]. In preparation for anaphase, this cohesion is disrupted at the S/G2 border by the parsylation of TRF1 by Tankyrase 1 [62]. In Tankyrase-deficient cells, telomere cohesion persists into anaphase, and this alteration results in telomere deprotection, sister chromatid fusions, and growth arrest [63]. This mechanism could also contribute, at least in part, to the lifespan-limiting activity of 3AB.

In summary, we report here that inhibitors of telomerase and poly(ADP-ribose) polymerase synergize to shorten the lifespan of pancreatic cancer cells. Additional studies will be required to elucidate the specific mechanisms responsible for the lifespan-limiting activity of 3AB, as well as the specific PARP family members involved. The acquired knowledge could potentially lead to the development of improved anti-telomerase therapies for patients afflicted with pancreatic cancer and other malignancies.

## MATERIALS AND METHODS

### Materials

All materials were purchased as previously described in Burchett *et al*., 2014 [6]. The palmitoyl-conjugated N3′ > P5′ thio-phosphoramidate GRN163L oligo (5′-palmitate-TAGGGTTAGACAA-NH_2_-3′) and mismatched oligo (5′-palmitate-TAGGTGTAAGCAA-NH_2_-3′; mismatches underlined) were provided by Geron Corporation (Menlo Park, CA, USA).

### Growth curves

The initial growth curves presented in Figure 1 were produced as we have previously done, except that cells were seeded at 5 × 10^5^ cells per 150 mm dish [6]. Cells were maintained in culture until the complete loss of the GRN163L-treated cultures or for at least 200 doublings done in the presence of the drugs.

L3.6 cells that became resistant to 1 μM GRN163L were subsequently cultivated at an increasing concentration of GRN163L. In a first step, the cells were cultivated in the presence of either 4 μM GRN163L or a maintenance dose of 1 μM GRN163L. In a second step, L3.6 cells that became resistant to 4 μM GRN163L were subsequently cultivated in the presence of either 10 μM GRN163L or a maintenance dose of 4 μM GRN163L. Drugs were added fresh every 2–3 days, with the two populations always receiving drugs in the same volumes of PBS vehicle. In curves testing the effects of combined exposure to GRN163L and 3AB, cells were grown in the presence of no drug, 3AB alone (3 mM, added twice a week), GRN163L alone (1 μM, added fresh every 2–3 days) or both drugs. Samples not receiving 3AB (dissolved in DMSO) or GRN163L (dissolved in PBS) were supplemented with the same corresponding volumes of DMSO and/or PBS vehicle.

### SA-β-galactosidase activity

Adherent cells were stained for the presence of SA-β-galactosidase activity as we have previously done [6].

### Western blot analysis

Western blot analysis of combined adherent and floating cells was done as we have done before [6]. Antibodies used against H2AX, **γ**H2AX (phospho-Ser139), PARP1, and actin were the same as previously described [6]. Also used were antibodies against Tankyrase (mouse monoclonal 19A449; IMGENEX), TRF1 (rabbit polyclonal ab1423; Abcam), and poly(ADP-ribose) chains (mouse monoclonal 3H2844; Santa Cruz).

### Flow cytometric analysis

Flow cytometric analysis of DNA content in combined adherent and floating cells was done as we have previously described [6].

### Telomere length analysis

Telomere length analysis was performed on DNA isolated from adherent cells as we have previously done [6], except that the probe was end-labeled [^32^P]-(CCCTAA)_4_ oligonucleotide.

### Detection of ALT by quantitative PCR

Detection of telomeric C-rich circles indicative of ALT was performed as we have previously described [6], following protocols developed by the Reddel group [54].

### Determination of relative telomerase activity

Relative telomerase activity in adherent cells was measured as we have done before [6]. Prior to measuring basal telomerase activity in cells that had previously been grown in the presence of GRN163L (e.g. GRN163L-resistant L3.6pl cells), cells were weaned off the drug for at least three weeks to eliminate traces of GRN163L prior to telomerase activity measurements.

### Determination of IC_50_ for GRN163L

Value of the IC_50_ for GRN163L was determined as we have done before [6]. To eliminate traces of GRN163L prior to IC_50_ determination, cells that were grown for at least three weeks in the absence of GRN163L prior to the experiments.

### Sequencing of the hTR gene

hTR sequences were amplified by PCR, starting with genomic DNA isolated from the 4 μM-resistant L3.6pl cells. Primers used were 5′-GACG CGGATCCGAGAGTCAGCTTGGCCAATC-3′ (BamHI site underlined) and 5′-GACGCGAATTCGGTGACG GATGCGCACGATC-3′ (EcoRI site underlined). PCR fragments were digested with BamHI plus EcoRI and inserted in the same two sites of pBluescript SK (-). Bacterial colonies were subjected to blue/white selection for the presence of an insert. Ten white colonies were picked and their plasmid sequenced using the M13 forward and reverse primers. No evidence of point mutations were detected in 10/10 independently cloned and sequenced hTR molecules.

### Expression of the hTERT gene

Total RNA from the two lines was reversed transcribed and subjected to PCR with primers specific for hTERT (5′-ACTCGACACCGTGTCACCTA-3′ and 5′-GTGACAGGGCTGCTGGTGTC-3′) or actin (5′-CGG GACCTGACTGACTACCT-3′ and 5′-CAGCACTGT GTTGGCGTACA-3′). RT-PCR was performed as we have done before [64]. Reactions were performed in either the presence or absence of reverse transcriptase.

### Level of hTR

Northern blot analysis was performed as we have done before [65]. Ten micrograms of total RNA from each of the two lines were resolved by electrophoresis and transferred to a nitrocellulose membrane. Membrane was probed with a random primed [^32^P]-labeled pTRC3 plasmid containing the hTR gene [66]. 18S ribosomal RNA was used to normalize signals for loading variation.

## SUPPLEMENTARY MATERIALS AND FIGURES


