# Supplementary material for: Inhibitors of telomerase and poly(ADP-ribose) polymerases synergize to limit the lifespan of pancreatic cancer cells
Source: Oncotarget. 2017 Jul 20;8(48):83754–67. doi: 10.18632/oncotarget.19410 (PMC5663552; doi:10.18632/oncotarget.19410)
Supplement: Supplementary file 1 [file oncotarget-08-83754-s001.pdf]

Inhibitors of telomerase and poly(ADP-ribose) polymerases synergize to limit the lifespan of pancreatic cancer cells

SUPPLEMENTARY MATERIALS

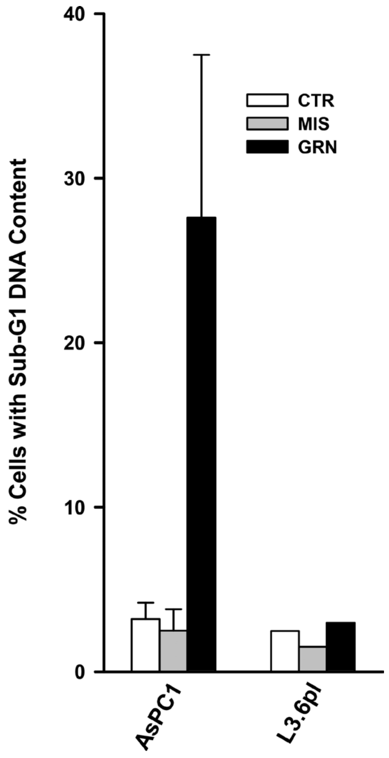

Supplementary Figure 1: Fraction of cells with sub-G1 DNA content. Floating and adherent cells were combined, stained with propidium iodide, and then analyzed for DNA content by flow cytometry. For both lines, measurements were made twice at a one week interval (Mean ± S.D., *n* = 2).

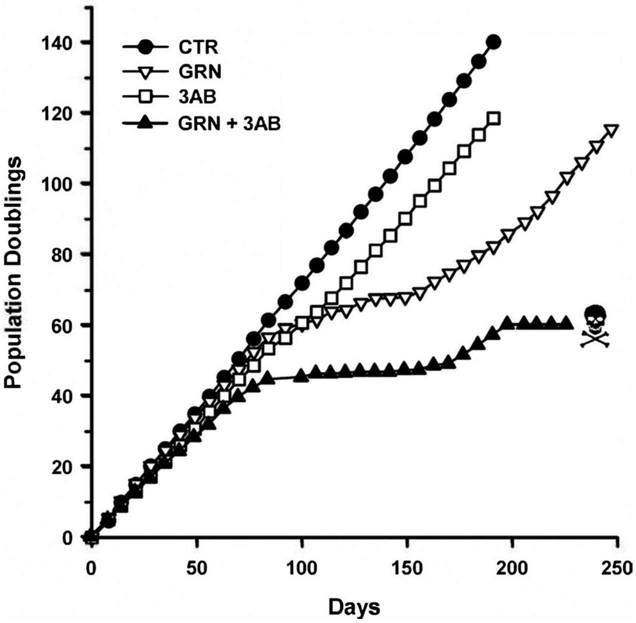

Supplementary Figure 2: 3AB synergize with GRN163L to limit the lifespan of the parental L3.6pl cells. Effects of 3AB and GRN163L on the lifespan of the parental L3.6pl cells. Parental L3.6pl cells were cultivated in the presence of no drug (CTR), 1 µM GRN163L (GRN), 3 mM 3AB (3AB), or 1 µM GRN163L + 3 mM 3AB (GRN + 3AB). Cells were given fresh GRN163L every 2–3 days and fresh 3AB twice a week. Growth curves show the number of population doublings achieved as a function of time. Skull and bone denotes loss of the culture. Cell pellets had been collected once every other week for telomere analysis, but these specimens were lost in an unforeseen accident. Thus, telomere size analysis could not be performed on any of the samples set aside from this purpose.
